# Supplementary material for: Twisted moiré photonic crystal enabled optical vortex generation through bound states in the continuum
Source: Nat Commun. 2023 Sep 27;14:6014. doi: 10.1038/s41467-023-41068-1 (PMC10533549; doi:10.1038/s41467-023-41068-1)
Supplement: Supplementary file 1 — Supplementary information [file 41467_2023_41068_MOESM1_ESM.pdf]

Supplementary Information for:

Twisted moiré photonic crystal enabled optical vortex generation through bound states in the continuum

Tiancheng Zhang[1 †], Kaichen Dong[2,3,4,5,6 * †], Jiachen Li[1,3], Fanhao Meng[1,3], Jingang Li[7], Sai Munagavalasa[8], Costas P. Grigoropoulos[7], Junqiao Wu[1,2,3], and Jie Yao[1,2,3 *]

Affiliations:
[1]Applied Science and Technology Graduate Group, University of California, Berkeley, CA, 94720, USA.
[2]Department of Materials Science and Engineering, University of California, Berkeley, CA, 94720, USA.
[3]Materials Sciences Division, Lawrence Berkeley National Laboratory, Berkeley, CA, 94720, USA.
[4]Tsinghua-Berkeley Shenzhen Institute, Tsinghua Shenzhen International Graduate School, Tsinghua University, Shenzhen, 518055, China.
[5]Institute of Data and Information, Tsinghua Shenzhen International Graduate School, Tsinghua University, Shenzhen, 518055, China
[6]Center of Double Helix, Tsinghua Shenzhen International Graduate School, Tsinghua University, Shenzhen, 518055, China
[7]Department of Mechanical Engineering, University of California, Berkeley, CA, 94720, USA.
[8]Department of Chemical and Biomolecular Engineering, University of California, Berkeley, CA, 94720, USA.
* Correspondence to: yaojie@berkeley.edu and dkc22@sz.tsinghua.edu.cn
† These authors contributed equally to the work

This PDF file includes:

A. Derivation of interlayer coupling coefficients in non-twist bilayer PhCs
B. Derivation of interlayer coupling coefficients in TBPCs
C. Derivation of the phase distribution of the emitted beam
D. The effect of changing twist angle and interlayer separation
E. The effect of changing the illumination position and direction
F. The comparison between analytical and numerical results
G. Optical vortex generation with a large twist angle

A. Derivation of interlayer coupling coefficients in non-twist bilayer PhCs

In general, we rely on the coupled mode theory to quantify the coupling between nearest neighbor (NN) disks, and then, in the same way of twisted bilayer graphene theory [1], the small twist angle allows the use of a continuum model for coupling coefficients calculations.

As mentioned in the main text, we start from a well-defined TE mode in a nano-disk [2]. We consider two coupled nano-disks of the same shape and material. When the two disks are placed close enough, the crosstalk between different cavity modes occurs, which is described by the coupled-mode theory:

$$\begin{cases} \dfrac{da_1(t)}{dt} = (i\omega_1 - \kappa_1)a_1 + ig_{12}a_2 \\ \dfrac{da_2(t)}{dt} = (i\omega_2 - \kappa_2)a_2 + ig_{21}a_1 \end{cases} \tag{S1}$$

where i, a, ω, and κ are the imaginary unit, the mode intensity, the eigenfrequency, and the decay rate, respectively. For identical disks, we have: $\omega_1 = \omega_2 = \omega_0$, $\kappa_1 = \kappa_2 = \kappa_0$. g_{12} and g_{21} are the coupling coefficient between the two TE modes. Without loss of generality, we set $g_{12} = g_{21} = g$ [2].

A monolayer photonic crystal (PhC) is constructed by combining these disks and form a planar periodic array. Here we consider a square lattice for simplicity, the major conclusions also work for a honeycomb lattice. In a square lattice, there are four NN disks for one disk. Thus, the equation of motion for each disk could be written as

$$\frac{da_j(t)}{dt} = (i\omega_0 - \kappa_0)a_j + \sum_{\boldsymbol{\delta}} \left(ig a_{j+\boldsymbol{\delta}}\right) \tag{S2}$$

where $\boldsymbol{\delta}$ is the site-to-site displacement with respect to disk j. In order to solve this equation, we use the transformation $a_j = \frac{1}{\sqrt{N}}\sum_{\boldsymbol{k}} \exp\left(-i\boldsymbol{k}\cdot\boldsymbol{r_j}\right) a_{\boldsymbol{k}}$, where r_j is the vector position of disk j and N is the total number of the disks. This leads to:

$$\frac{1}{\sqrt{N}}\sum_j \exp\left(i\boldsymbol{k}'\cdot\boldsymbol{r}_j\right) a_j = \frac{1}{\sqrt{N}}\sum_j \exp\left(i\boldsymbol{k}'\cdot\boldsymbol{r}_j\right) \frac{1}{\sqrt{N}}\sum_{\boldsymbol{k}} \exp\left(-i\boldsymbol{k}\cdot\boldsymbol{r}_j\right) a_{\boldsymbol{k}}$$
$$= \frac{1}{N}\sum_{\boldsymbol{k}}\sum_j \exp\left[i(\boldsymbol{k}'-\boldsymbol{k})\cdot\boldsymbol{r}_j\right] a_{\boldsymbol{k}} = \sum_{\boldsymbol{k}} \delta(\boldsymbol{k}'-\boldsymbol{k}) a_{\boldsymbol{k}} = a_{\boldsymbol{k}'} \tag{S3}$$

So, we have $a_{\boldsymbol{k}} = \frac{1}{\sqrt{N}}\sum_j \exp\left(i\boldsymbol{k}\cdot\boldsymbol{r}_j\right) a_j$, where $a_{\boldsymbol{k}}$ represents the mode intensity of the guided resonances with wave vector $\boldsymbol{k}$ (herein coined as mode $\boldsymbol{k}$ for simplicity). With Fourier transform conducted, equation (S2) is transformed into [2]:

$$\frac{da_{\boldsymbol{k}}}{dt} = \left(i\omega_0 - \kappa_0 + ig\sum_{\boldsymbol{\delta}} \exp(-i\boldsymbol{k}\cdot\boldsymbol{\delta})\right) a_{\boldsymbol{k}} \tag{S4}$$

Here, we notice that there is only one term that is proportional to $a_{\boldsymbol{k}}$ on the right side of the equation, meaning that each guided resonance does not couple to other guided

resonances with different wave vectors. We note that the interaction between disks is calculated using the spatial integral of the product of two terms: one mode and the complex conjugation of the other mode. As such, this interaction term will become zero if the integral in different periods have different phases.

In non-twisted bilayer PhCs systems, we now have two set of disks: a_1, and a_2, representing disks in PhC 1 and PhC 2, respectively. Here, we take disk a_{1j} as an example. By only considering NN interlayer interactions [1], we have:

$$\frac{da_{1j}}{dt} = (i\omega_0 - \kappa_0)a_{1j} + \sum_{\boldsymbol{\delta}_1}\left(ig_{intra}a_{1(j+\delta)}\right) + ig_{inter}a_{2j} \tag{S5}$$

where g_{inter} denotes the coupling coefficient between two nearest neighbor disks in PhC 1 and PhC 2. We use g_{intra} to denote the intralayer coupling coefficient to prevent confusion.

Analogous to the monolayer case, we can define $a_{1\boldsymbol{k_1}}$, and $a_{2\boldsymbol{k_2}}$ from $a_{j1} = \frac{1}{\sqrt{N}}\sum_{\boldsymbol{k}} \exp\,(-i\boldsymbol{k}\cdot\boldsymbol{r_{1j}})\, a_{1\boldsymbol{k_1}}$, and $a_{j2} = \frac{1}{\sqrt{N}}\sum_{\boldsymbol{k}} \exp\,(-i\boldsymbol{k}\cdot\boldsymbol{r_{2j}})\, a_{2\boldsymbol{k_2}}$, we have:

$$\begin{cases} a_{1\boldsymbol{k}_1} = \dfrac{1}{\sqrt{N}}\displaystyle\sum_j \exp\left(i\boldsymbol{k}_1\cdot\boldsymbol{r}_{1j}\right) a_{1j} \\ a_{2\boldsymbol{k}_2} = \dfrac{1}{\sqrt{N}}\displaystyle\sum_j \exp\left(i\boldsymbol{k}_2\cdot\boldsymbol{r}_{2j}\right) a_{2j} \end{cases} \tag{S6}$$

Taking these equations to equation (S5), we have:

$$\frac{da_{1\boldsymbol{k}_1}}{dt} = \frac{1}{\sqrt{N}}\sum_j \exp\left(i\boldsymbol{k}_1\cdot\boldsymbol{r}_{1j}\right)\frac{da_{1j}}{dt}$$

$$= \left(i\omega_0 - \kappa_0 + ig_{intra}\sum_{\boldsymbol{\delta}}\exp(-i\boldsymbol{k}\cdot\boldsymbol{\delta})\right)a_{1\boldsymbol{k}_1} + ig_{inter}a_{2\boldsymbol{k}_1} \tag{S7}$$

From equation (S7) we noticed that the guided resonance in PhC 1 with wave vector $\boldsymbol{k}_1$ can only couple to the guided resonance in PhC 2 with the same wave vector. For at-Gamma bound state in the continuum (BIC) mode in PhC 1, it will only couple to at-Gamma BIC mode in PhC 2. The above claims explain the Fig. 2a in the main text, where the interlayer coupling intensity is all zero except the at-Gamma point.

B. Derivation of interlayer coupling coefficients in TBPCs

We introduce a minute twist between the two stacking PhC layers and consider the twisted bilayer photonic crystal (TBPC) case. By only considering NN interlayer interactions [1], we have:

$$\frac{da_{1j}}{dt} = (i\omega_0 - \kappa_0)a_{1j} + \sum_{\boldsymbol{\delta}_1}\left(ig_{intra}a_{1(j+\delta)}\right) + ig_{inter}(\boldsymbol{l})a_{2(j+\boldsymbol{l})} \tag{S8}$$

where $\boldsymbol{l}$ means the displacement from disk $a_{1,j}$ to its closet disk in PhC 2, and $g_{inter}(\boldsymbol{l})$ denotes the coupling coefficient between these two disks.

Analogous to the non-twist bilayer case, we can define $a_{1\boldsymbol{k_1}}$, and $a_{2\boldsymbol{k_2}}$ from $a_{j1} = \frac{1}{\sqrt{N}}\sum_{\boldsymbol{k}} \exp\left(-i\boldsymbol{k}\cdot\boldsymbol{r_{1j}}\right) a_{1\boldsymbol{k_1}}$, and $a_{j2} = \frac{1}{\sqrt{N}}\sum_{\boldsymbol{k}} \exp\left(-i\boldsymbol{k}\cdot\boldsymbol{r_{2j}}\right) a_{2\boldsymbol{k_2}}$. Taking equation (S6) into equation (S8), we have:

$$\frac{da_{1\boldsymbol{k}_1}}{dt} = \frac{1}{\sqrt{N}}\sum_j \exp\left(i\boldsymbol{k}_1\cdot\boldsymbol{r}_{1j}\right)\frac{da_{1j}}{dt}$$

$$= \left(i\omega_0 - \kappa_0 + ig_{intra}\sum_{\boldsymbol{\delta}}\exp(-i\boldsymbol{k}\cdot\boldsymbol{\delta})\right)a_{1\boldsymbol{k}_1} + \frac{i}{\sqrt{N}}\sum_j \exp\left(i\boldsymbol{k}_1\cdot\boldsymbol{r}_{1j}\right) g_{inter}(\boldsymbol{l})a_{2(j+\boldsymbol{l})} \quad \text{(S9)}$$

Then we expand $a_{2(j+\boldsymbol{l})}$ in terms of $a_{2\boldsymbol{k_2}}$ to the second term

$$\frac{i}{\sqrt{N}}\sum_j \exp\left(i\boldsymbol{k}_1\cdot\boldsymbol{r}_{1j}\right) g_{inter}(\boldsymbol{l})a_{2(j+\boldsymbol{l})}$$

$$= \frac{i}{N}\sum_j \exp\left(i\boldsymbol{k}_1\cdot\boldsymbol{r}_{1j}\right) g_{inter}(\boldsymbol{l})\sum_{\boldsymbol{k}_2}\exp\left(-i\boldsymbol{k}_2\cdot\left(\boldsymbol{r}_{1j,a}+\boldsymbol{l}\right)\right)a_{2\boldsymbol{k}_2}$$

$$= \sum_{\boldsymbol{k}_2}\zeta(\boldsymbol{k}_1,\boldsymbol{k}_2)a_{2\boldsymbol{k}_2} \quad \text{(S10)}$$

where

$$\zeta(\boldsymbol{k}_1,\boldsymbol{k}_2) = \frac{i}{N}\sum_j \exp\left(i(\boldsymbol{k}_1-\boldsymbol{k}_2)\cdot\boldsymbol{r}_{1j}\right)\cdot\exp(-i\boldsymbol{k}_2\cdot\boldsymbol{l})\cdot g_{inter}(\boldsymbol{l}) \quad \text{(S11)}$$

Taking equation (S10) into equation (S9), we have the coupling equation for mode $\boldsymbol{k}_1$ as:

$$\frac{da_{1\boldsymbol{k}_1}}{dt} = \left(i\omega_0 - \kappa_0 + ig_{intra}\sum_{\boldsymbol{\delta}}\exp(-i\boldsymbol{k}\cdot\boldsymbol{\delta})\right)a_{1\boldsymbol{k}_1} + \sum_{\boldsymbol{k}_2}\zeta(\boldsymbol{k}_1,\boldsymbol{k}_2)a_{2\boldsymbol{k}_2} \quad \text{(S12)}$$

From equation (S12), we notice that $\zeta(\boldsymbol{k}_1,\boldsymbol{k}_2)$ is the coupling coefficient between mode $\boldsymbol{k}_1$ in PhC 1 and mode $\boldsymbol{k}_2$ in PhC 2. By introducing a twist structure and breaking the short-range periodicity, g_{inter} function will change at difference spatial positions, making $\zeta(\boldsymbol{k}_1,\boldsymbol{k}_2)$ finite even if $\boldsymbol{k}_1 \neq \boldsymbol{k}_2$.

We can calculate this coupling coefficient by equation (S11). Within the square superlattice around the AA site, we find $\boldsymbol{l} = \boldsymbol{\theta}\times\boldsymbol{r}_{1j}$. For the BIC mode in PhC 2, we have $\boldsymbol{k}_2 = \boldsymbol{0}$, and we denote $\zeta(\boldsymbol{k}_1) = \zeta(\boldsymbol{k}_1,\boldsymbol{0})$ in short. Specifically speaking, $\zeta(\boldsymbol{0})$ denotes the coupling coefficient between the BIC modes in the two layers. Therefore, equation (S11) can be presented as:

$$\zeta(\boldsymbol{k}_1) = \frac{i}{N}\sum_j \exp\left(i\boldsymbol{k}_1\cdot\boldsymbol{r}_{1j}\right)\cdot g_{inter}\left(\boldsymbol{\theta}\times\boldsymbol{r}_{1j}\right) \quad \text{(S13)}$$

By defining a new g function as: $g\left(\boldsymbol{r}_{1j}\right) = g_{inter}\left(\boldsymbol{\theta}\times\boldsymbol{r}_{1j}\right)$, we can transform equation (S13) to:

$$\zeta(\boldsymbol{k}_1) = \frac{i}{N}\sum_j \exp(i\boldsymbol{k}_1 \cdot \boldsymbol{r}_{1j}) \cdot g(\boldsymbol{r}_{1j}) \tag{S14}$$

Using the continuum model [3], we change the sum into an integration over a single superlattice with area of S_c:

$$\zeta(\boldsymbol{k}_1) = \frac{i}{S_c}\iint \exp(i\boldsymbol{k}_1 \cdot \boldsymbol{r}_1) \cdot g(\boldsymbol{r}_1) d^2 r_1 \tag{S15}$$

Then we calculate the g function using the coupled-mode theory [4]:

$$g = \frac{\omega \iiint \delta\, \epsilon_1 e_1^* e_2 dv}{2 \iiint \epsilon_1 |e_1|^2} \tag{S16}$$

where e stands for the electric-field distribution of the interested mode, and ϵ stands for the dielectric constant distribution. Then we take this back to equation (S15) and we can get the coupling coefficient.

E-field distribution and dielectric constant distribution are both critical to our calculation process using the coupled mode theory. Here, the E-field distribution of the TE mode in a silicon disk was numerically calculated by COMSOL Multiphysics. Otherwise specified, the diameter and height of the silicon nanodisk are 450 nm and 220 nm. The corresponding mode profile is shown in Supplementary Fig. 1.

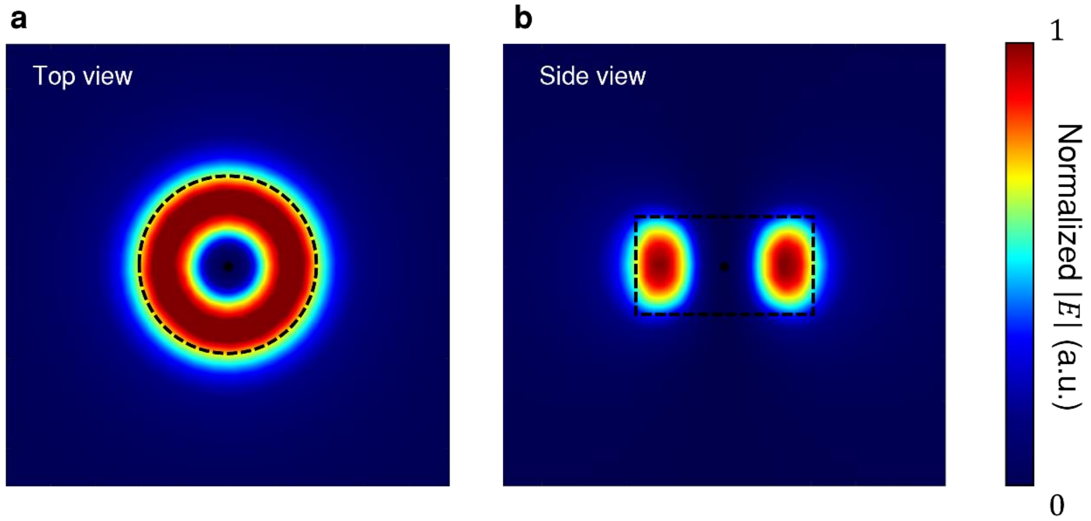

Supplementary Fig. 1 | The top view (a) and side view (b) of the TE mode in a Si nano-disk solved by COMSOL Multiphysics. The black dashed lines indicate the Si nano-disk profile.

Note that in the simulation for Supplementary Fig. 1, the dielectric constant (ϵ) distribution is:

$$\begin{cases} \epsilon(x,y,z) = n^2 & x^2 + y^2 \le \left(\frac{d}{2}\right)^2, |z| \le \frac{h}{2} \\ \epsilon(x,y,z) = 1 & elsewhere \end{cases} \tag{S17}$$

where n, d, and h are the refractive index of silicon, diameter, and height of the nano-disk, respectively. Without loss of generality, the refractive index of silicon is set to be 3.47.

With the above numerical e, we can take equation (S16) and equation (S17) into equation (S15) and get the coupling coefficient between a guided resonances $\boldsymbol{k}_1$ in PhC 1 and the BIC mode in PhC 2. The representative results when the lattice constant $a_s = 700$ nm are shown in Supplementary Fig. 2a. Note here that the coupling intensity is real since $g(\boldsymbol{l})$ function is isotropic.

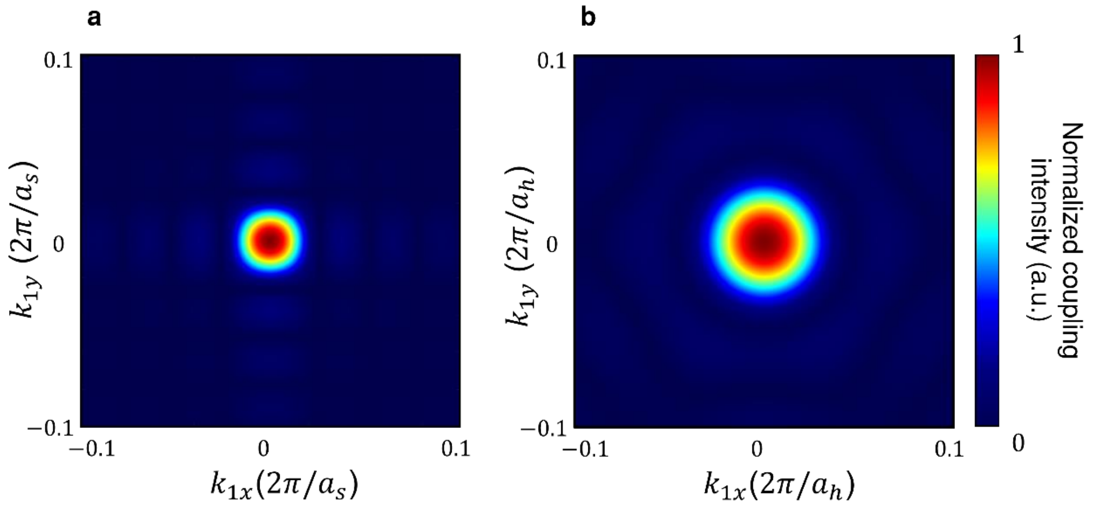

Supplementary Fig. 2 | Interlayer coupling intensity for a square lattice (a) and a honeycomb lattice (b). Here, the interlayer separation is 300 nm and the twist angle is 1.5°.

Supplementary Fig. 2 shows that the coupling intensity is negligible when $\boldsymbol{k}_1$ is far away from the Gamma point, and when $\boldsymbol{k}_1$ is close to the Gamma point, the coupling intensity is nearly isotropic. Hence, if we choose a circular loop around the Gamma point, the modes on the loop will be excited with almost identical intensity and the same phase. To further verify our conclusion, we moved on and theoretically analyzed the TBPCs with honeycomb lattices with the lattice constant of $a_h = 1000$ nm (Supplementary Fig. 2b), finding that they are subject to similar behaviors as those in square lattices.

C. Derivation of the phase distribution of the emitted beam

In order to derive the phase information of the emission from the coupling intensity in Supplementary Fig. 2, we start from the temporal coupled mode theory (TCMT) main equations:

$$\begin{cases} \frac{dA(\boldsymbol{k}_1)}{dt} = [i\omega(\boldsymbol{k}_1) - \kappa(\boldsymbol{k}_1)]A(\boldsymbol{k}_1) + \zeta(\boldsymbol{k}_1)A_{BIC} \cdot \exp{(i\omega_{BIC}t)} \\ \boldsymbol{s}^- = \boldsymbol{D}A \end{cases} \tag{S18}$$

where A is the resonance amplitude in PhC 1 with specific in-plane wave vector $\boldsymbol{k}_1$, $\omega(\boldsymbol{k}_1)$ the mode's resonant frequency, $\kappa(\boldsymbol{k}_1)$ the decay rate due to radiative loss. A_{BIC} and ω_{BIC} are the amplitude and the resonant frequency of the BIC mode in PhC 2, respectively. $\zeta(\boldsymbol{k}_1)$ is the coupling intensity between mode $\boldsymbol{k}_1$ in PhC 1 and the BIC mode in PhC 2. $\boldsymbol{s}^- = (E_s^- \quad E_p^-)^T$ is the outgoing wave amplitude, and $\boldsymbol{D} = (d_s \quad d_p)^T$ is the coupling coefficient between the resonance and the outgoing plane wave. The subscript $s(p)$ here represent the $s(p)$-polarized portion. Note here that equation (S18) is a bit different from the TCMT main equations considered in the transmission situations because the ingoing wave amplitude is zero, and mode $\boldsymbol{k}_1$ in PhC 1 is excited by the BIC mode in PhC 2 since other low-Q modes dissipate much faster than the BIC mode.

We calculate the projection of $\boldsymbol{s}^-$ and $\boldsymbol{D}$ onto the x-y plane, from equation (S18) we know:

$$\boldsymbol{s}^- = \begin{pmatrix} E_x^- \\ E_y^- \end{pmatrix} = A \begin{pmatrix} d_x \\ d_y \end{pmatrix} \tag{S19}$$

Apparently, this is the Jones vector of the outgoing plane wave. When the monolayer PhC is invariant under the operation $C_2^z T$, d_x and d_y can be chosen to be real number simultaneously [5]. Thus, we denote d_x and d_y as:

$$\begin{pmatrix} d_x \\ d_y \end{pmatrix} = \sqrt{d_x^2 + d_y^2} \cdot \begin{pmatrix} \cos\big(\theta(\boldsymbol{k}_1)\big) \\ \sin\big(\theta(\boldsymbol{k}_1)\big) \end{pmatrix} \tag{S20}$$

where $\theta(\boldsymbol{k}_1) = \arg{(d_x + i d_y)}$ is the angle of the polarization vector. We divide $\boldsymbol{s}^-$ in the basis of left-hand circular polarization (LCP), $|L\rangle = \frac{1}{\sqrt{2}}\begin{pmatrix} 1 \\ +i \end{pmatrix}$, and right-hand circular polarization (RCP), $|R\rangle = \frac{1}{\sqrt{2}}\begin{pmatrix} 1 \\ -i \end{pmatrix}$:

$$\boldsymbol{s}^- = A\sqrt{d_x^2 + d_y^2} \cdot \begin{pmatrix} \cos\big(\theta(\boldsymbol{k}_1)\big) \\ \sin\big(\theta(\boldsymbol{k}_1)\big) \end{pmatrix} = \frac{A\sqrt{d_x^2 + d_y^2}}{\sqrt{2}} e^{-i\theta(\boldsymbol{k}_1)}|L\rangle + \frac{A\sqrt{d_x^2 + d_y^2}}{\sqrt{2}} e^{i\theta(\boldsymbol{k}_1)}|R\rangle \tag{S21}$$

From equation (S18), we know that $A\sqrt{d_x^2 + d_y^2} = \zeta(\boldsymbol{k}_1) \cdot \frac{A_{BIC}\sqrt{d_x^2 + d_y^2}}{i(\omega_{BIC} - \omega) + \kappa}$. Thus, the emitted field $|E_{out}\rangle = \boldsymbol{s}^-$ will be:

$$|E_{out}\rangle = K\zeta(\boldsymbol{k}_1)e^{-i\theta(\boldsymbol{k}_1)}|L\rangle + K\zeta(\boldsymbol{k}_1)e^{i\theta(\boldsymbol{k}_1)}|R\rangle \tag{S22}$$

Here, $K = \frac{A_{BIC}\sqrt{d_x^2 + d_y^2}}{\sqrt{2}[i(\omega_{BIC} - \omega) + \kappa]}$ is nearly a constant if we choose a circular loop around the Gamma point [6]. $\zeta(\boldsymbol{k}_1)$ has been calculated by equation (S15), which is also nearly a constant in the circular loop around the Gamma point as shown in Supplementary Fig. 2. Thus, $K\zeta(\boldsymbol{k}_1)$ shall be not able to introduce another winding phase factor near the

Gamma point. So, we can notice from equation (S22) that the phase of the LCP (RCP) portion of the emission is $\theta_{LCP} = -\theta$ ($\theta_{RCP} = \theta$), which is exactly the opposite polarization angle (or the same as the polarization angle). The states of polarization host a vortex near the Gamma point. Thus, the TBPC system is able to generate phase-vortex emission in the far-field, which have the opposite (same) topological order with the BIC. For the TBPC system, all the above modes that are interconnected by the twist-enabled coupling mechanism and generate an optical vortex emission are inseparable and can be considered as one mode, herein coined as "moiré mode" for simplicity.

For better illustration, we also show a schematic for the vortex generation in Supplementary Fig. 3. In real space, if we circulate around the beam center, the polarization of the beam will always be close to linear polarization, with the polarization angle changing from 0° to $l \cdot 360°$, where l is the topological order of the BIC. Thus, this trajectory in the real space correspond to the equator of a Poincare sphere in the parameter space, as shown in Supplementary Fig. 3 [7]. Since it is on the equator of a Poincare sphere, we can also regard the SOPs as the combination of an LCP portion and an RCP portion with the same amplitude but with different phases. Specifically speaking, $|E_{out}\rangle = e^{-i\theta(\boldsymbol{k}_1)}|L\rangle + e^{i\theta(\boldsymbol{k}_1)}|R\rangle$.

Therefore, for a trajectory circulating the beam center in the real space, its SOPs will rotate by $\theta(\boldsymbol{k}_1)$ while the phases of its LCP portion and RCP portion will gain an extra phase $\mp\theta(\boldsymbol{k}_1)$. If the trajectory forms a loop in the real space, the phases of its LCP portion and RCP portion will increase by $\mp l \times 2\pi$, forming an optical vortex structure.

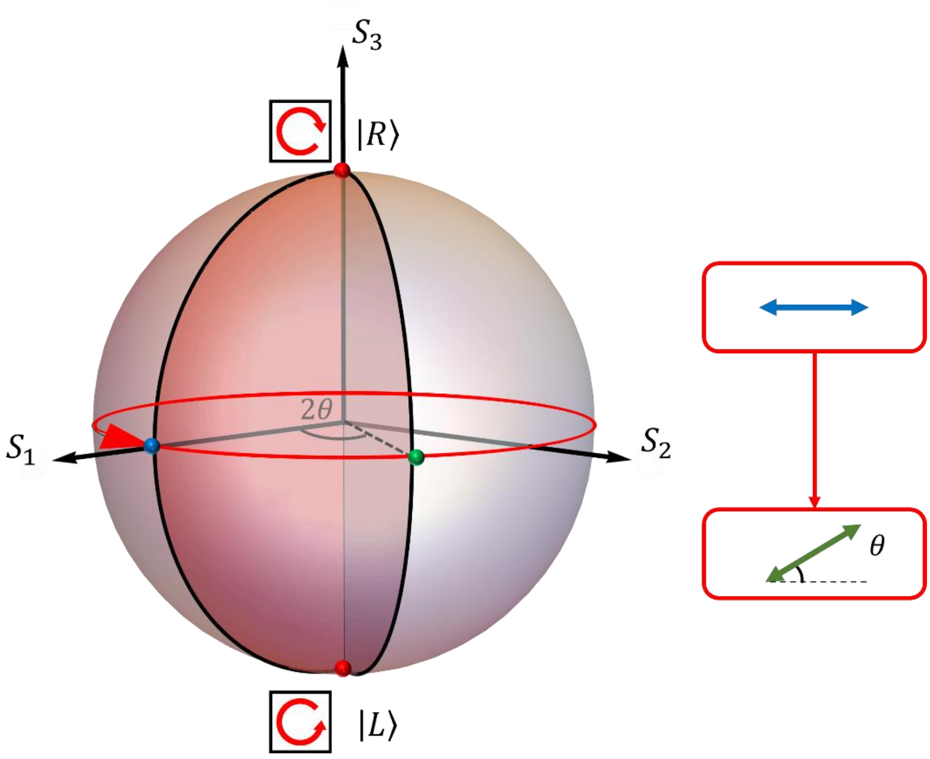

Supplementary Fig. 3 | Schematic for the vortex generation. Different linear states of polarization (double-headed arrows) correspond to different positions on the equator of the Poincaré sphere. S1, S2 and S3 are the first, second and third Stokes parameters [8], respectively. The blue (green) double headed arrows correspond to the linear SOPs at the blue (green) dots on the equator of the Poincaré sphere.

As predicted in previous papers [5], the quantized topological charge of the at-Gamma BIC of a square lattice is l =1. We profile the optical vortex generated by a TBPC with square lattice in the far field by numerical simulation (Supplementary Fig. 4). The lattice constant of the monolayer is 700 nm. The interlayer separation and twist angle between the two layers are 400 nm and 1.2°, respectively. We notice that the topological order of the LCP and RCP portion are exactly l_{LCP} =-1 and l_{RCP} =1, respectively, which perfectly match our theoretical prediction.

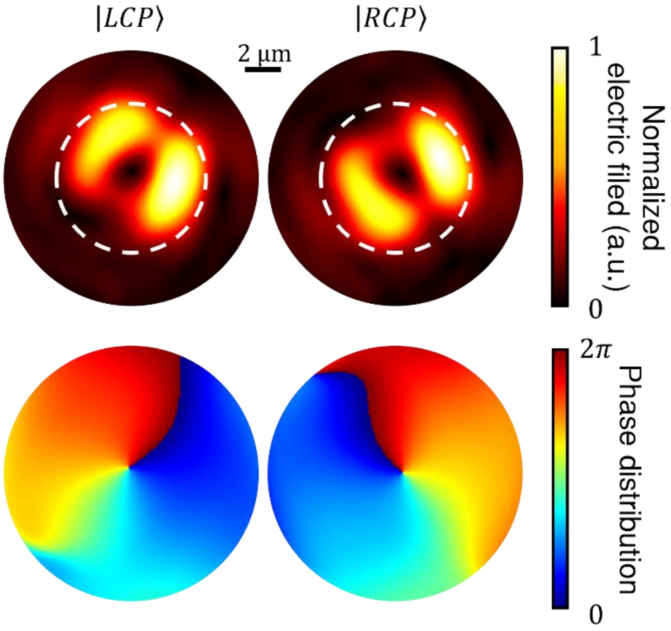

Supplementary Fig. 4 | Optical vortex generated in a TBPC system with square lattices. The upper panels depict the normalized amplitude of the LCP and RCP portions of the far-field emission, while the lower panels are the phase distribution of the corresponding upper panels in dashed white circles.

D. The effect of changing twist angle and interlayer separation

Although the topological order of the emitted optical vortex is immune to the change in either interlayer separation or twist angle, the Q factor of the moiré mode is strongly dependent on those two geometric factors. As proved in previous papers [2], the interlayer coupling intensity goes down as the interlayer separation increases, which further reduces the energy transferring rate from at-Gamma BIC to the free space. Meanwhile, a smaller twist angle leads to weaker interlayer coupling. Thus, the Q factor of the moiré mode can be enhanced by increasing the interlayer separation and decreasing the twist angle. As shown in Supplementary Fig. 5, we calculated the frequency and Q-factor corresponding to the parameters chosen in Fig. 4a in the main text. When the interlayer separation and twist angle are changed, the frequency of the moiré mode merely changes, because the frequency of the moiré mode is determined by the frequency of the BIC mode but not the interlayer separation or the twist angle. Meanwhile, the Q-factor changes from 1.0×10^3 to 6.8×10^3 when the interlayer separation changes from 200 nm to 350 nm, and changes from 4.1×10^3 to 2.4×10^3 when the twist angle changes from 1.0° to 2.5°.

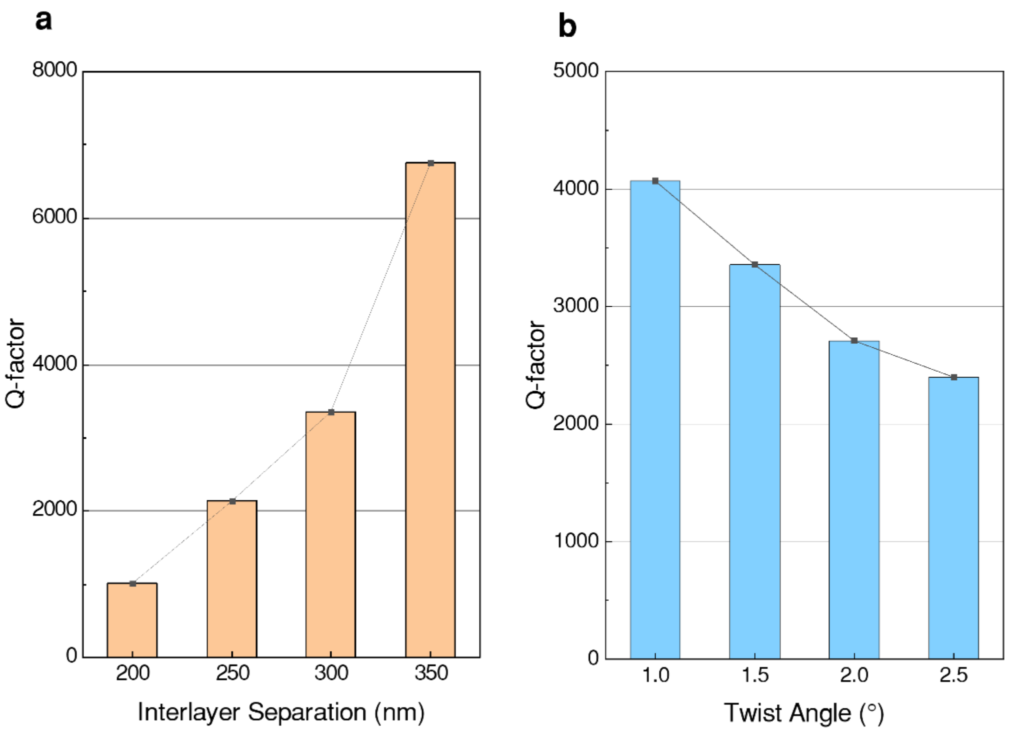

Supplementary Fig. 5 | Q-factor with different interlayer separations and twist angles. a, Q-factor with different interlayer separations when twist angle is 1.5°. **b,** Q-factor with different twist angles when interlayer separation is 300 nm.

E. The effect of changing the illumination position and direction

The existing optical vortex generators rely on accurate alignment of the position or the direction of the incident beam. However, since the optical vortex emission in TBPC is generated by the BIC modes, it is independent of the position and incident angle of the incident beam. Here, we perform a numerical calculation with the same conditions as Fig. 3 but with different positions and incident angles for the incident beam. The results in Supplementary Fig. 6 justify that changing the incident position or incident angle does not affect the emitted optical vortex. The vortex emission from a TBPC system is insensitive to fabrication error, thermal expansion, and incident beam, which is promising as a future platform for stable optical vortex emission.

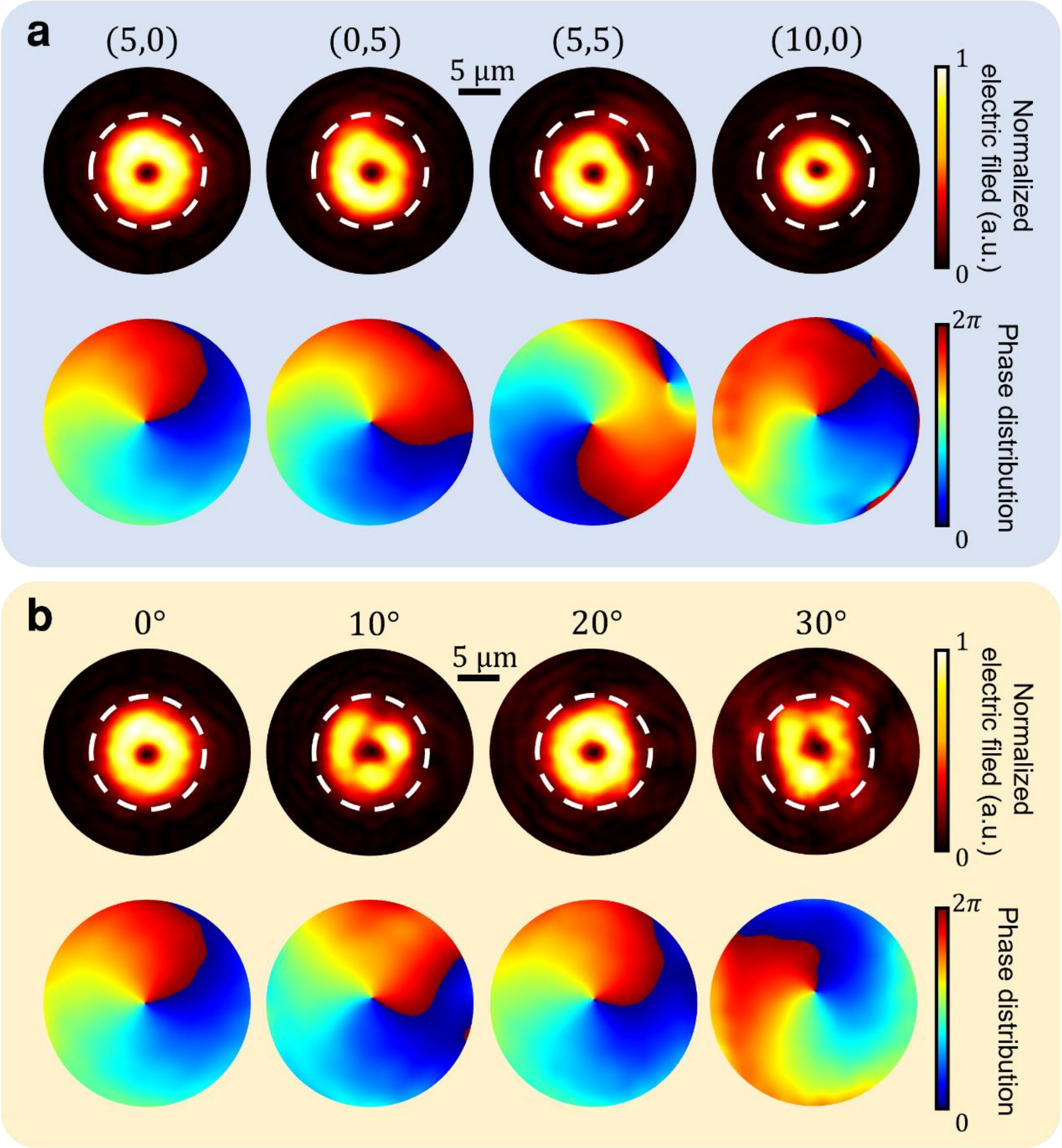

Supplementary Fig. 6 | Optical vortices generation as functions of various incident beam center positions and incident angles. a, Normalized intensity and phase distribution with different incident beam center positions: (5 μm, 0 μm), (0 μm, 5 μm), (5 μm, 5 μm), and (10 μm, 0 μm). The incident angle is fixed at 0°, and the FWHM of the incident gaussian beam is 3 μm. **b,** Normalized intensity and phase distribution with different incident angles: 0°, 10°, 20°, and 30°. The incident beam center position is fixed at (5 μm, 0 μm).

F. The comparison between analytical and numerical results

Quantitative comparison between the analytical results and simulation results are important for verifying the theoretical model. In order to make a comparison between the analytical and numerical results, we analytically and numerically calculate the divergence angle of the vortex beam in the far field as a function of the twist angle.

The divergence angle is a key feature of the vortex beam and also critical for the future vast applications of the generated optical vortex [9-11]. Also, different from the topological order of the vortex or the eigenfrequency, which is mainly defined by the single layer properties, the divergence angle of the vortex beam is directly connected to the interlayer coupling mechanism. Comparing the analytically and numerically calculated divergence angle will confirm the reliability of our theoretical model.

First, the analytical calculation is conducted as follows. According to the coupled mode theory, the BIC in PhC 2 will couple to the guided resonances $\boldsymbol{k}_1$ in PhC 1, and the corresponding coupling intensity $\zeta(\boldsymbol{k}_1)$ is:

$$\zeta(\boldsymbol{k}_1) = \frac{i}{S_c} \iint \exp(i\boldsymbol{k}_1 \cdot \boldsymbol{r}_1) \cdot g(\boldsymbol{r}_1) d^2 r_1 \qquad (S23)$$

where $g(\boldsymbol{r}_1)$ is the coupling intensity of interlayer nearest neighbor disks at $\boldsymbol{r}_1$ point. Based on the simulation of a single particle and single layer photonic crystal, we can calculate the interlayer coupling strength $\zeta(\boldsymbol{k}_1)$.

The profile of the function $\zeta(\boldsymbol{k}_1)$ is plotted in Supplementary Fig. 7 (left column), and the range of $\zeta(\boldsymbol{k}_1)$ increases when the twist angle goes up. We note that the guided resonances $\boldsymbol{k}_1$ will further couple to the far field with frequencies and in-plane wave vectors matching the plane waves in free space. As a result, when the range of $\zeta(\boldsymbol{k}_1)$ increases, the range of in-plane wave vectors of the plane waves in free space also increases, leading to a larger divergence angle of the generated optical vortex beam. Therefore, we can deduce the divergence angle analytically by evaluating the $\zeta(\boldsymbol{k}_1)$ function.

Secondly, by simulating the beam profile in Lumerical FDTD, we can directly get the divergence angle, as shown in Supplementary Fig. 7 (right column).

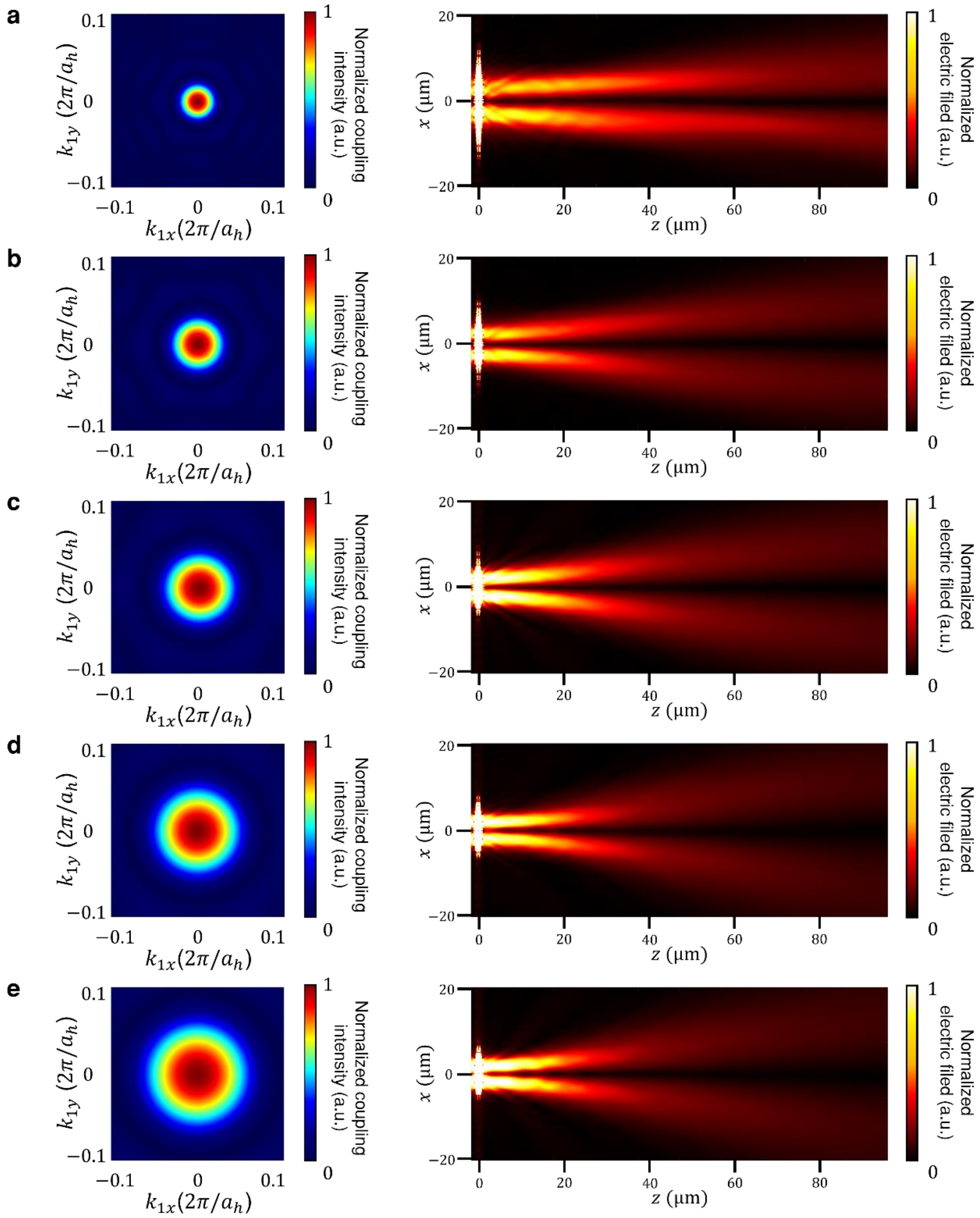

Supplementary Fig. 7 | Analytically calculated normalized interlayer coupling intensity (left column) and numerically simulated xz-plane beam profile (right column) as functions of twist angles. The twist angles are 1.0° (a), 1.5° (b), 2.0° (c), 2.5° (d), and 3.0° (e), respectively. The twisted bilayer photonic crystal (TBPC) is centered at $z = 0$ plane in the right column.

With the above results, we compare the analytically calculated and simulated divergence angles in Supplementary Fig. 8. The numerically calculated divergence angle has the same trend as the analytical results: the divergence angle increases at larger twist angles. The slight discrepancy could be further decreased by considering

higher orders of coupling in the theory model and use finer meshes in the numerical simulation.

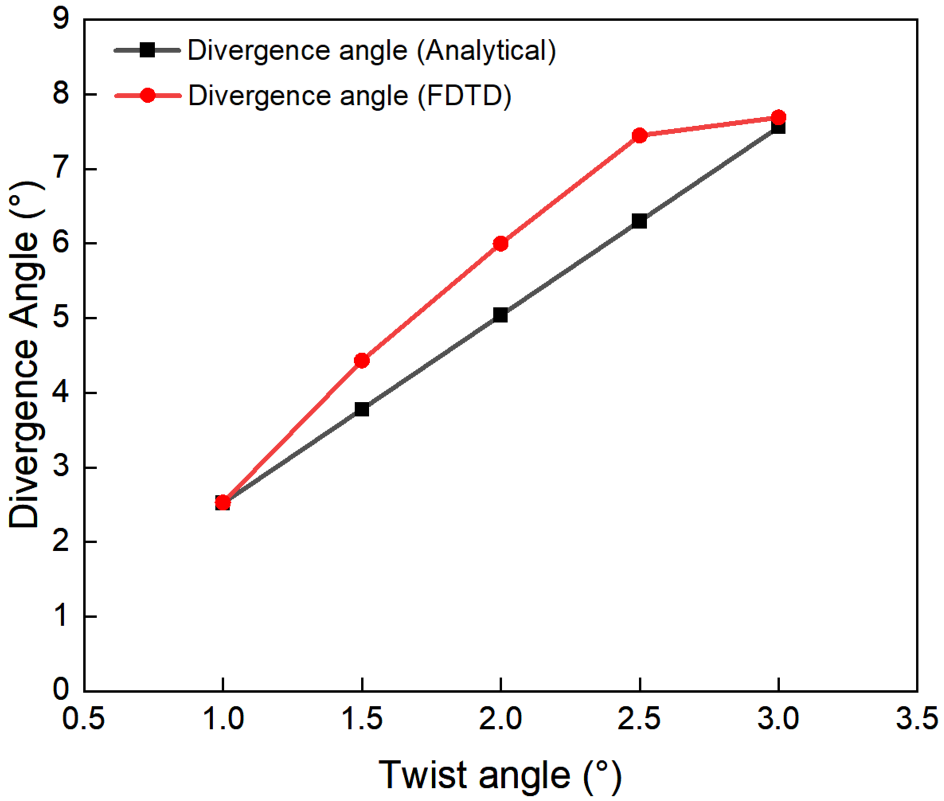

Supplementary Fig. 8 | Comparison between analytical calculated and simulated divergence angles.

G. Optical vortex generation with a large twist angle

As shown in Supplementary Fig. 9, we further simulate the radiated beam profile from the TBPC systems with larger twist angles. We find that with the increase of twist angle, the quality of the emitted optical vortex gets worse. If we further increase the twist angle (> 8°), the radiated beam will be chaotic and there is no obvious vortex feature.

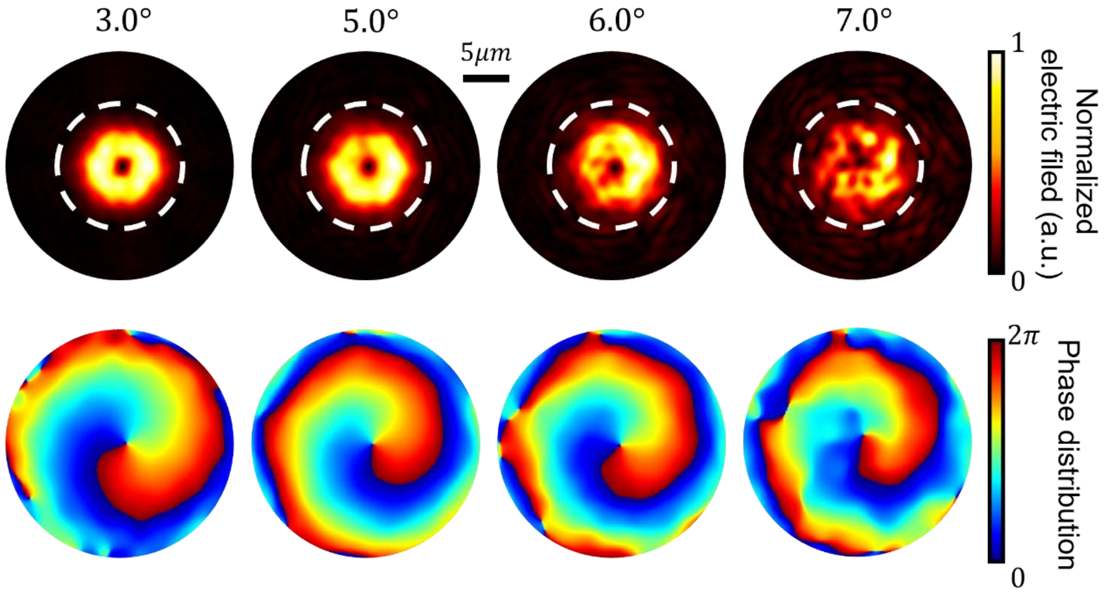

Supplementary Fig. 9 | Optical vortices generation as functions of large twist angles. The bottom panels are the corresponding phase distributions of the top panels in dashed white circles.

This is because when the twist angle is small, the coupling between the BIC mode in one photonic crystal slab and the guided resonances in the other photonic crystal slab is weak, and thus the BIC mode decays slowly. However, when the twist angle increases, the coupling between the BIC mode and the guided resonances becomes stronger, leading to a much faster decay rate of the BIC mode as well as a lower Q factor (Supplementary Fig. 5).

In our FDTD simulation, the TBPC system is excited with a pulsed beam. When the Q factor of the BIC mode is high at smaller twist angles, all unwanted guided resonances will dissipate much faster than the BIC mode. Thus, after about 5 ps, the radiation by the BIC mode (i.e., the optical vortex emission) will gradually stand out against the chaotic background as all unwanted low-Q modes vanish (Fig. 5). On the contrary, when the Q factor of the BIC mode is low at larger twist angles, both the BIC mode and the guided resonances dissipate quickly. Thus, the generated beam will be a mixture of the radiation from both the BIC mode and the guided resonances, resulting in a low-quality optical vortex.

References

1. Bistritzer, Rafi, and Allan H. MacDonald. "Moiré bands in twisted double-layer graphene." *Proceedings of the National Academy of Sciences* 108.30 (2011): 12233-12237. https://doi.org/10.1073/pnas.1108174108
2. Dong, Kaichen, et al. "Flat Bands in Magic-Angle Bilayer Photonic Crystals at Small Twists." *Physical review letters* 126.22 (2021): 223601.

https://doi.org/10.1103/PhysRevLett.126.223601
3. Mennucci, Benedetta. "Polarizable continuum model." *Wiley Interdisciplinary Reviews: Computational Molecular Science* 2.3 (2012): 386-404. https://doi.org/10.1002/wcms.1086
4. Haus, Hermann A., and Weiping Huang. "Coupled-mode theory." *Proceedings of the IEEE* 79.10 (1991): 1505-1518. https://doi.org/10.1109/5.104225
5. Zhen, Bo, et al. "Topological nature of optical bound states in the continuum." *Physical review letters* 113.25 (2014): 257401. https://doi.org/10.1103/PhysRevLett.113.257401
6. Wang, Bo, et al. "Generating optical vortex beams by momentum-space polarization vortices centred at bound states in the continuum." *Nature Photonics* 14.10 (2020): 623-628. https://doi.org/10.1038/s41566-020-0658-1
7. Chipman, Russell A., Wai Sze Tiffany Lam, and Garam Young. *Polarized light and optical systems*. CRC press, 2018.
8. Stokes, George Gabriel. "On the composition and resolution of streams of polarized light from different sources." *Transactions of the Cambridge Philosophical Society* 9 (1851): 399.
9. Chung, Hyeongju, et al. "Generation of E-band metasurface-based vortex beam with reduced divergence angle." *Scientific Reports* 10.1 (2020): 1-8. https://doi.org/10.1038/s41598-020-65230-7
10. Vallone, Giuseppe, et al. "General theorem on the divergence of vortex beams." *Physical Review A* 94.2 (2016): 023802. https://doi.org/PhysRevA.94.023802
11. Ma, Hongyu, et al. "Broadband vortex beams generation with narrow divergence angle using polarization insensitive metasurface." *IEEE Access* 8 (2020): 218062-218068. https://doi.org/10.1109/ACCESS.2020.3042236
